# Trace element distribution in soils and food in intensively cultivated tropical areas of Mato Grosso, Brazil

**DOI:** 10.1038/s41598-026-41252-5

**Published:** 2026-04-09

**Authors:** Daisy Rickli Binde, Milton Ferreira de Moraes, Stephan M. Haefele, Martin R. Broadley

**Affiliations:** 1 Department of Education, Federal Institute of Mato Grosso, Barra do Garças, MT 78605-099 Brazil; 2 Department of Agronomy, Graduate Program in Tropical Agriculture, Faculty of Agronomy and Animal Science, Federal University of Mato Grosso, Cuiabá, MT 78060-900 Brazil; 3 Department of Sustainable Soils and Crops, Rothamsted Research, West Common, Harpenden, Hertfordshire AL5 2JQ UK; 4 Rothamsted Research, West Common, Harpenden, Hertfordshire AL5 2JQ UK

**Keywords:** Geochemical background, Cerrado biome, Tropical soils, Bioaccumulation, Heavy metals, Sustainable agriculture, Biogeochemistry, Ecology, Ecology, Environmental sciences

## Abstract

The Cerrado biome of Mato Grosso, a major agricultural region in Brazil, requires continuous soil management and fertility correction. Assessing trace elements (TEs) in soils and crops is essential to understand their dynamics and potential environmental and food safety implications. This study evaluated the concentrations of thirteen TEs (As, Cd, Co, Cr, Cu, Fe, Mn, Mo, Ni, Pb, Se, and Zn) in soils and agricultural products from representative areas of the region. A total of 84 soil samples (0–0.20 m), 84 soybean grains (*Glycine max* L.), 48 corn grains (*Zea mays* L.), 12 popcorn grains (*Zea mays everta*), and 8 pasture samples (*Urochloa ruziziensis* R. Germ. & C.M. Evrard) were analyzed. Following aqua regia extraction, TEs were quantified using inductively coupled plasma mass spectrometry (ICP-MS) and optical emission spectrometry (ICP-OES). Descriptive, inferential, and multivariate analyses, including bioaccumulation factor estimation, were applied. Overall, the soils showed no evidence of severe contamination, and the analyzed food products indicated a low immediate risk to food safety when assessed against regulatory limits. Elevated As, Cr, and Fe concentrations in soils were mainly associated with the natural geochemical background, whereas Zn, Cd, Cu, and Mn were partly related to the use of agricultural inputs. Integrated statistical and geoenvironmental analyses provided support for the discussion of possible trace-element sources in tropical agricultural systems.

## Introduction

Plants play a fundamental role as a source of minerals in the human diet, including trace elements (TEs), which are essential for meeting daily nutritional needs. The deficiency of certain elements in the diet can result in malnutrition and have negative impacts on public health^1–3^. On the other hand, the bioaccumulation of TEs has become an increasingly relevant environmental concern, intensified by industrialization and the expansion of intensive agriculture. The excessive use of fertilizers, soil amendments, and pesticides, combined with inadequate soil management practices, accelerates the introduction and dispersion of these pollutants in the environment, increasing the risks of soil and water contamination and, consequently, their entry into the food chain^4,5^. The daily intake of potentially toxic TEs by the population occurs largely through the consumption of vegetables, as widely consumed foods such as rice, corn, wheat, potatoes, and soybeans can serve as exposure pathways^6,7^. The impact of TE bioaccumulation varies depending on the plant species and may compromise food safety^6,8^.

Among the most relevant agricultural crops in the Brazilian context, corn and soybeans stand out for their widespread cultivation and importance in both human and animal nutrition. Corn provides about 20% of global calories, being rich in vitamins, minerals, and biomass. It is widely consumed in developing countries and used in the food industry^9,10^. Soybeans, on the other hand, are notable for their high protein content, richness in amino acids, fiber, and essential minerals, making them a viable alternative to animal protein and a raw material for oils and processed foods^11,12^. Besides these essential crops for human nutrition, pasture quality also deserves attention, as it is directly related to animal health and, consequently, to the consumption of animal-derived products. High TE concentrations in the soil can contaminate forage, affecting milk and meat quality, while their deficiency can compromise animal health and productivity^13,14^.

Beyond their nutritional value, the importance of these agricultural crops is closely tied to their production regions, such as the Cerrado, which is crucial for agricultural production in Mato Grosso (MT), one of the world’s leading grain producer^15^. The region’s agricultural relevance is directly linked to the biome’s characteristics, which include arable soils that have been extensively exploited since the 1970s. These soils often require chemical corrections and intensive management to maintain fertility^16^. Given this, analyzing TEs in these soils and in agricultural crops from these regions is essential to understanding the impacts of agricultural practices on environmental and food quality. The study of these elements allows for the assessment of potential changes resulting from input use, contributing to more sustainable and safer management practices.

Despite the Cerrado’s agricultural significance, there remains a considerable gap in studies focusing on the presence of TEs in the region’s agricultural crops, particularly in soybeans and corn. Most research on TEs in crops worldwide has been conducted in areas with a history of industrial or mining contamination, as studied by Antoniadis et al.^17^, Blanco et al.^18^, Duan et al.^19^, and Xu et al.^20^. In Brazil, studies such as Schwalbert et al.^21^, which investigated contamination in vineyards, and Cândido et al.^22^, which analyzed Pb-contaminated tropical soils, are available. However, research addressing the bioaccumulation of these elements in areas of intensive agricultural production, such as the Cerrado, remains scarce.

Several studies have demonstrated that agricultural inputs can be sources of Cd and Pb contamination, due to the natural occurrence of Cd in phosphate rocks and Pb in limestone rocks used in production^23–25^. However, the impact of these products on food quality is still underexplored in traditional agricultural production areas. The study by Corguinha et al.^26^ was one of the few to investigate the presence of As, Cd, and Pb in Brazilian crops, including soybeans and corn collected in Itiquira (MT), a region with prolonged phosphate fertilizer use in the state of Mato Grosso. These findings highlight the need for systematic assessments in Cerrado agricultural areas, where continuous input use may intensify the bioaccumulation of potentially toxic TEs, compromising environmental quality and food safety.

The Mato Grosso Cerrado stands out as one of the world’s largest food-producing regions and is highly dependent on agricultural inputs; therefore, the study of trace elements (TEs) in the region’s soils is essential to ensure food quality and promote sustainable production. The bioaccumulation or deficiency of these elements in food directly affects nutritional quality and human health, making the assessment of their concentrations in different crops critically important. Against this backdrop, this study adopts an integrated, data-driven approach that combines pedological and geological information with statistical analyses, as well as comparisons with current environmental legislation and regional scientific studies^27,28^, to support the interpretation of the possible sources of TEs. Accordingly, the objectives of this study are to analyze the distribution of TEs (As, Cd, Co, Cr, Cu, Fe, Mn, Mo, Ni, Pb, Se, and Zn) in soils from productive regions of the Mato Grosso Cerrado and to investigate the transfer of these elements to soybean grains (*Glycine max*, L), corn (*Zea mays*, L), popcorn *(Zea mays everta*), and pasture (*U. ruziziensis*, R.Germ. and C.M.Evrard).

## Materials and methods

### The territory of mato grosso

The Cerrado is the predominant biome in the agricultural region of Mato Grosso, covering an area of 354,823 km^2^^29^. The region’s soils require intensive management for the production of soybean, maize, beef, and cotton, which, as the main agricultural activities, directly influence the environmental dynamics of the biome^30^; IMEA^31^. According to the Köppen classification, the region exhibits a strong north-south precipitation gradient, with an Am climate prevailing in the north and an Aw climate covering most of the territory, particularly in the Cerrado and Pantanal biomes. The Aw climate is characterized by a well-defined dry season, annual precipitation ranging from 1,400 mm (Pantanal) to 2,300 mm (Planalto dos Parecis), and average temperatures between 18 °C and 35 °C^32^.

### Sampling

**Fig. 1:**
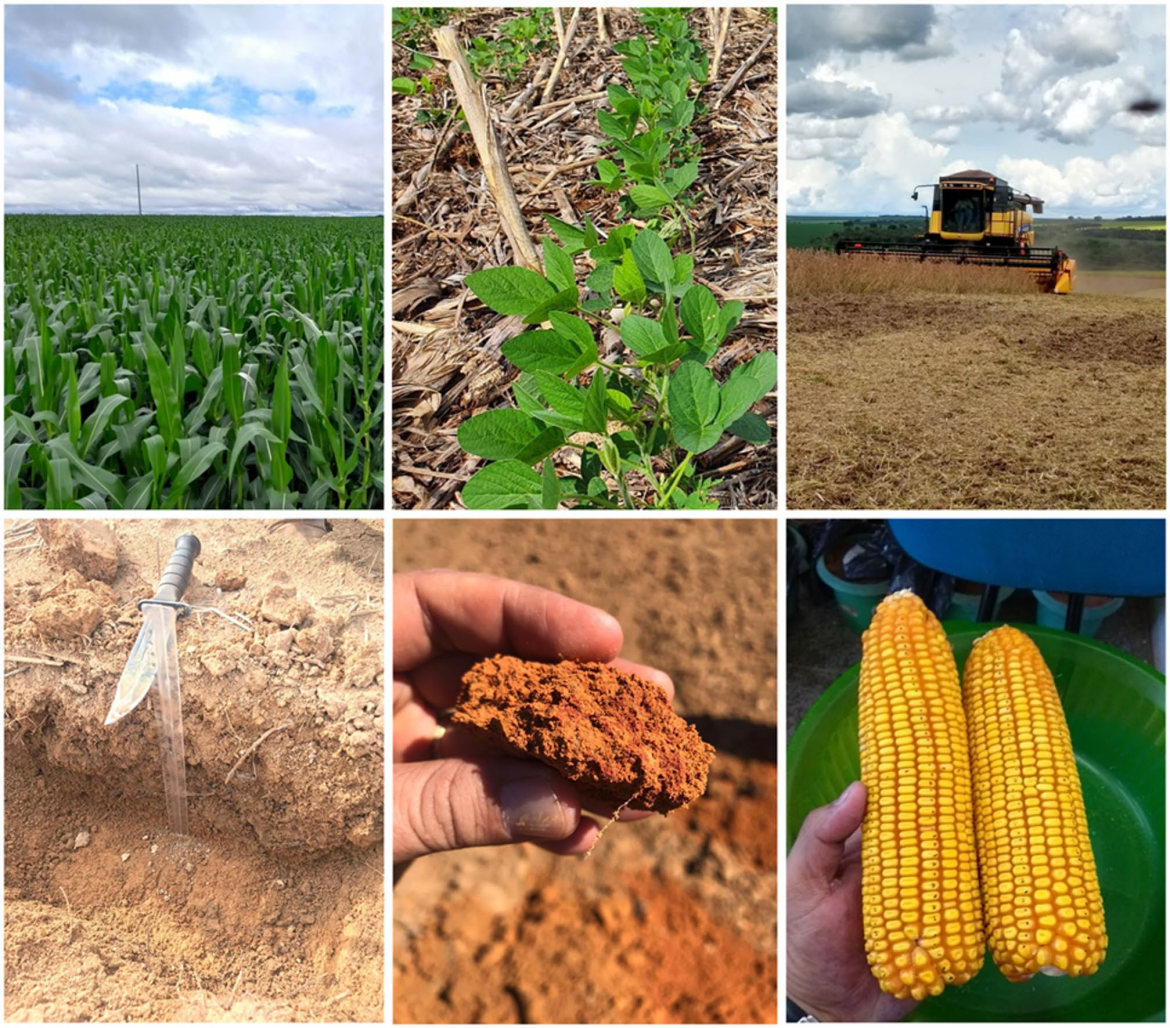
Representative images of the study area in the Cerrado region of Mato Grosso, Brazil, showing agricultural systems, soils, and crops sampled for trace element analysis.

**Fig. 2:**
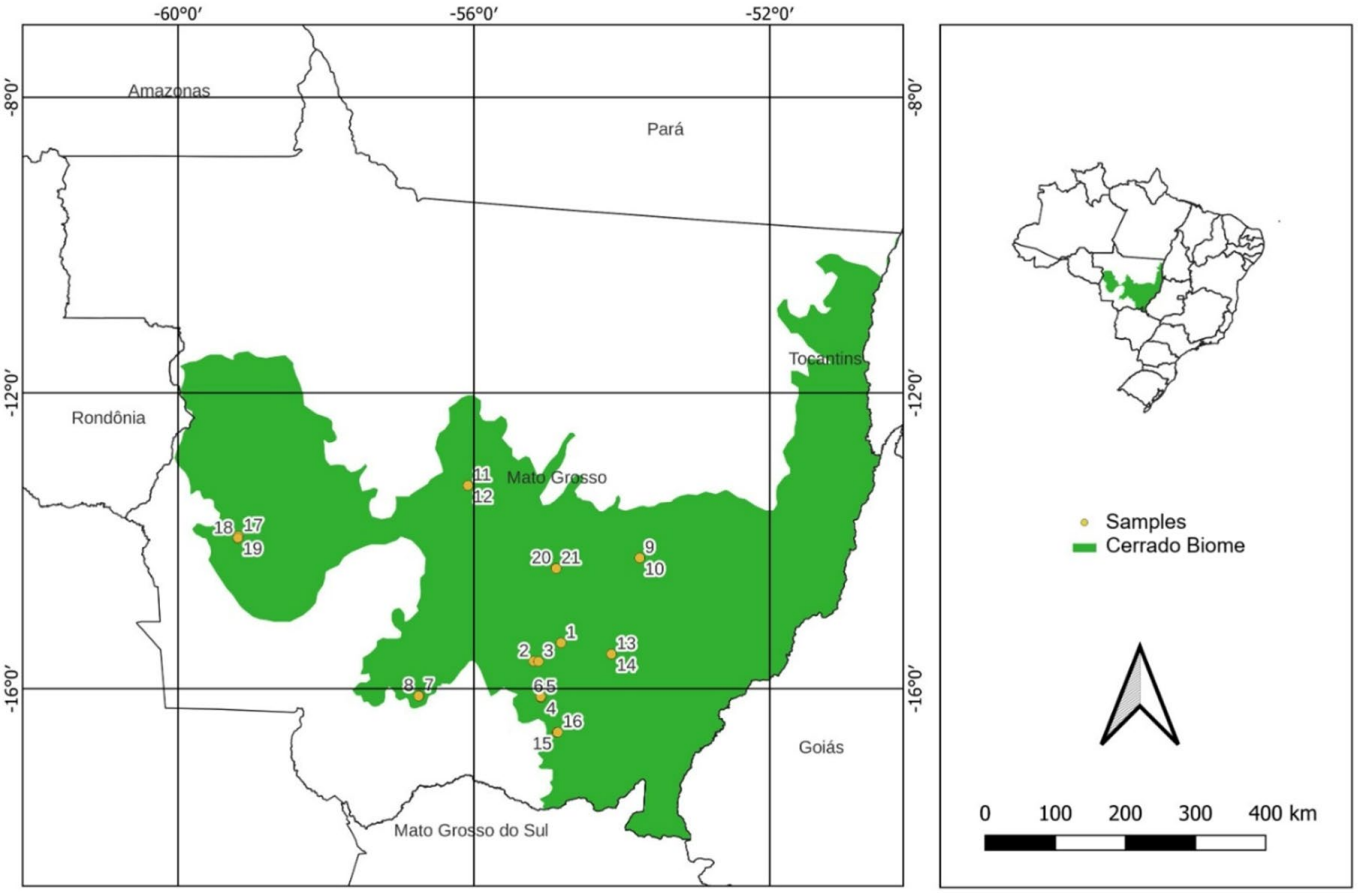
Location of the 21 samples from the main commercial soybean-growing areas in the state of Mato Grosso, Brazil.

The sampled areas correspond to commercial agricultural farms representative of typical production systems of the Mato Grosso Cerrado (Fig. 1), with sampling conducted in collaboration with the Association of Soybean and Maize Producers of Mato Grosso (APROSOJA). Soil and crop samples were collected from productive plots, taking into account farm operational logistics, crop availability across growing seasons, and the adoption of conventional agricultural practices, including the use of commercial inputs. Sampling was conducted in plots distributed across 21 georeferenced locations (Fig. 2), encompassing the main soybean-producing regions of the Cerrado in Mato Grosso^31^. These sites are described in Table 1, along with georeferencing data. Sampling was performed in quadruplicate, totaling 84 soil samples prior to the 2021 harvest. In the same areas, agricultural product samples were collected: 84 soybean grain samples (2021 harvest), 48 maize grain samples, 12 popcorn maize samples, and 8 pasture samples (second harvest of the same year). Differences in sample numbers among agricultural products reflect the actual dynamics of production systems between growing seasons.

In each municipality, at least two georeferenced sampling sites were selected. At each site, a 3 × 3 grid was established, totaling nine points with a regular spacing of 50 m between them. Four points were randomly selected for collection. At each point, a composite soil sample was collected, consisting of four subsamples at a depth of 0–20 cm. Soil classes were determined according to the Brazilian Soil Classification System^33^, and their corresponding World Reference Base (WRB-FAO) soil groups were identified for international comparison. While environmental data, geological provinces, and lithologies were obtained from IBGE^34^. In the laboratory, the material was air-dried, crushed, sieved (< 2 mm), and stored for analysis. Agricultural product samples were collected at harvest from the same areas as the soil samples, covering an area of 32.4 m^2^ (5 m of two central rows per plot). Samples were cleaned and dried at 60 °C until reaching constant weight.

**Table 1:** Characterization of the sampled locations in the Cerrado, including soil types, geological province (Pg), lithology (Lit), municipality, and geographic coordinates - latitude (Lat) and longitude (Long) - as well as the specification of the 2nd crop, since the 1st crop was exclusively soybean in all locations.

Location	Municipality	Soil (SiBCS) - Soil (WRB)	Gp	Lit	2ª Crop	Lat (S)	Long (O)
1	Campo verde	RL - Ferralsol	Cen	Sd	Pastagem	15° 22’ 50.54’’	54° 49’ 17.68’’
2	Campo verde	RYL - Ferralsol	Cen	Sd	Pastagem	15° 37’ 46.75’’	55° 11’ 29.19’’
3	Campo verde	RYL - Ferralsol	Cen	Sd	Milho	15° 37’ 57.59’’	55° 7’ 41.39’’
4	Juscimeira	RL - Ferralsol	Cen	Sdc	Milho	16° 7’ 12.18’’	55° 5’ 35.16’’
5	Juscimeira	RL - Ferralsol	Cen	Sd	Milho	16° 7’ 0.11’’	55° 5’ 40.52’’
6	Juscimeira	RL - Ferralsol	Cen	Sd	Milho	16° 6’ 31.36’’	55° 5’ 49.43’’
7	Poconé	RL - Ferralsol	Toc	Mt	–	16° 5’ 47.84’’	56° 44’ 22.95’’
8	Poconé	RYL- Ferralsol	Toc	Mt	–	16° 6’ 0.23’’	56° 44’ 52.19’’
9	Paranatinga	XC - Cambisol	Toc	Sd	Milho	14° 13’ 50.19’’	53° 45’ 27.43’’
10	Paranatinga	XC - Cambisol	Toc	Sd	Milho	14° 13’ 50.19’’	53° 45’ 27.43’’
11	Lucas do Rio Verde	QR - Arenosol	Cen	Is	Milho	13° 14’ 59.38’’	56° 4’ 51.53’’
12	Lucas do Rio Verde	QR - Arenosol	Cen	Is	Milho	13° 15’ 0.8’’	56° 4’ 52.56’’
13	Primavera do Leste	RL- Ferralsol	Cen	Sdc	Milho	15° 32’ 5.19’’	54° 8’ 24.5’’
14	Primavera do Leste	RL- Ferralsol	Cen	Sd	Milho	15° 31’ 59.57’’	54° 8’ 23.11’’
15	Rondonópolis	RL - Ferralsol	Cen	Sdc	Milho	16° 35’ 35.24’’	54° 52’ 43.94’’
16	Rondonópolis	RL - Ferralsol	Cen	Sdc	Milho	16° 35’ 28.28’’	54° 52’ 3.03’’
17	Campos de Júlio	RL- Ferralsol	Cen	Sd	Pipoca	13° 55’ 58’’	59° 11’ 4.57’’
18	Campos de Júlio	RL - Ferralsol	Cen	Sd	Pipoca	13° 57’ 47.94’’	59° 11’ 31.17’’
19	Campos de Júlio	RL - Ferralsol	Cen	Sd	Pipoca	13° 57’ 30.36’’	59° 11’ 25.92’’
20	Planalto da Serra	XC - Cambisol	Toc	Sd	–	14° 22’ 9.69’’	54° 53’ 31.84’’
21	Planalto da Serra	XC - Cambisol	Toc	Sd	–	14° 22’ 25.38’’	54° 53’ 17.95’’

Sd, sedimentary; Sdc, conglomerate sedimentary; Mt, metamorphic; Is, igneous-sedimentary; XC, hapludox cambisol; RL, red latosol; RYL, red-yellow latosol; QR, quartzarenic neosols; Cen, cenozoic; To, tocantins. Geographic coordinates are provided in the WGS84 reference system.

### Laboratory analyses

The physicochemical analyses (Table 2) were performed as described by Prado et al.^35^. For the quantification of TEs, soil samples were ground in a planetary ball mill (Retsch PM400, Retsch GmbH, Germany) to a particle size below 0.1 μm and subsequently digested with aqua regia solution in an open system^36^. Each digestion batch for soil samples included three replicates - one every 15 samples - two standards, and two blanks. The standards used for method validation were ISE 962/WEPAL and ERM-CC141 (Clayey Soils). Dried agricultural product samples were ground and digested in a microwave system (CEM MARS 6) using a closed system^37^. In each batch of 40 samples, three repetitions were included - one every 10 samples - along with a standard and a blank, both in duplicate. The standard used for grains was the certified wheat flour sample 1567b (NIST), while for pasture, the IPE 881 (wheat straw - *Triticum aestivum*, L.) was used. The elements Mo, Ni, Cr, Co, Pb, Cd, Se, and As were quantified using inductively coupled plasma mass spectrometry (ICP-MS, NexION 300X), while Ca, Cu, Fe, K, Mg, Mn, Na, P, S, and Zn were analyzed by inductively coupled plasma optical emission spectrometry (ICP-OES, Agilent 5900 SVDV).

**Table 2:** Soil chemical and granulometric properties in the sampled municipalities.

Municipality	OM (g Kg^− 1^)	TOC (g Kg^− 1^)	pH H_2_O	CEC (cmol_c_ kg^− 1^)	Clay (g kg^− 1^)	Silt (g kg^− 1^)	Sand (g kg^− 1^)
Campo Verde	31.10	18.02	6.1	8.9	552.7	166.8	280.4
Campos de Júlio	28.74	16.68	5.9	8.0	527.8	140.1	332.1
Juscimeira	23.93	13.88	5.8	7.2	230.5	111.1	658.5
Lucas do Rio Verde	28.64	16.59	5.6	8.3	427.1	180.3	392.6
Paranatinga	20.64	12.09	5.7	7.8	337.5	143.9	518.7
Planalto da Serra	24.04	14.00	5.5	9.2	399.0	214.6	386.4
Poconé	48.33	28.00	5.5	17.6	473.8	173.4	352.8
Pontes Lacerda	27.08	15.71	5.6	7.5	309.5	122.3	568.2
Rondonópolis	29.29	17.18	5.7	8.7	635.3	173.3	191.5

OM, organic matter; TOC, total organic carbon; pH H_2_O, pH in water; CEC, cation exchange capacity.

The concentrations of TEs were determined using calibration curves obtained from certified standard solutions. The average recovery rates in soil for the analyzed elements ranged between 90% and 110%, except for Mo, which varied between 80% and 90%. In grains, the average recovery rates for Ca, Cu, Fe, K, Mn, Mo, and Na ranged from 90% to 100%, while the other elements showed values between 70% and 90%. For pasture samples, the average recovery rates were 45% to 50% for As, Se, and Mo, 73% for Al, 95% for Ni, and between 50% and 65% for the remaining elements. The detection limits (DLs) in mg kg^− 1^ for the elements in soil were: As (0.004), Cd (0.002), Co (0.01), Cr (0.08), Cu (0.21), Fe (29.7), Mn (1.76), Mo (0.01), Ni (0.18), Pb (0.09), Se (0.002), Zn (0.64); for grains: As (0.0004), Cd (0.0004), Co (0.003), Cr (0.05), Cu (0.10), Fe (0.54), Mn (0.008), Mo (0.02), Ni (0.04), Pb (0.03), Se (0.0003), Zn (0.13); and for pasture: As (0.001), Cd (0.0009), Co (0.007), Cr (0.09), Cu (0.38), Fe (2.73), Mn (0.03), Mo (0.02), Ni (0.07), Pb (0.06), Se (0.0007), and Zn (1.83). Quality control was performed using blanks and internal standards to ensure that the results were within ± 2 standard deviations (sd). Additionally, one sample out of every 10 was reanalyzed to check the repeatability of the results, with an acceptable variation of 5–10%, depending on the element.

Calibration was performed with certified standard solutions, using 7 to 8 standards in ICP-OES (linear or quadratic fit, depending on the element) and 6 standards in ICP-MS (linear fit). Accuracy was verified using certified reference materials, and samples exceeding the calibration limit were diluted and reanalyzed. Drift corrections were applied during the analyses: in ICP-OES, using yttrium (Y) as an internal standard and/or quality control solution, and in ICP-MS, using indium (In) as an internal standard. Samples analyzed by ICP-OES were measured in Synchronous Vertical Dual View mode, capturing both axial and radial plasma views in a single reading, with spectral corrections applied for elements such as Na, Cr, and Ni. In ICP-MS, Kinetic Energy Discrimination and Dynamic Reaction Cell methods were employed, using gases like NH₃ and O_2_ to eliminate polyatomic and spectral interferences.

### Statistical analysis of data

A descriptive statistical analysis was performed, considering means, medians, and coefficients of variation. The Shapiro-Wilk test (1965) indicated a lack of normality, leading to the adoption of a non-parametric approach. The TE concentration results in soils and crops were compared with current legislation^38–40^ and literature data, including the Quality Reference Values (QRV) proposed by Silva et al.^28^, although this proposal has not yet been approved. To visualize the soil data distribution and identify exceedances of the Prevention Value (PV), a boxplot was constructed using the Contamination Factor (CF), calculated as the ratio between the TE concentration in soil and its respective PV^17^. Data were log-transformed (log10) for better interpretation, and a red dashed line was included in the graph to indicate the PV reference limit.

The Kruskal-Wallis test^41^ and Dunn’s test (with Bonferroni adjustment)^42^ were applied to verify significant differences (*p* < 0.05) in TE concentrations of soils and crops among different locations. Pasture samples were excluded from these comparisons due to the limited sample size (*n* = 8) and the associated analytical uncertainties. Additionally, soil samples were analyzed for differences between lithologies and soil types, while for geological provinces, the Mann-Whitney test^43^ was used. The Kruskal-Wallis and Dunn tests were also employed to compare the analyzed crops. The Spearman correlation^44^ between TE concentrations in soil and their respective concentrations in grains was assessed. Based on these correlations, a distance dendrogram (d = 1 − |ρ|) was constructed, and the Ward.D2 clustering method (*p* < 0.05) was applied.

The transfer efficiency of TEs was quantified using the Bioaccumulation Factor (BAF), calculated as the ratio between the TE concentrations in grains and those in soil (Ali et al. 2019)^17^. BAF values were log-transformed (natural logarithm) and analyzed using Welch’s ANOVA. Data distribution was visualized using boxplots, and BAF values were classified into accumulation categories: very low (< 0.1), low (0.1–0.5), moderate (0.5-1.0), high (1.0–5.0), very high (5.0–20.0), and hyperaccumulation (≥ 20.0). The classifications were analysed by crop, location, and TE, aiming to explore spatial patterns and differentiation between crops.

All statistical analyses were conducted using RStudio (version 2022.12.0) and Microsoft Excel (Version 2402 Build 16.0.17328.20124). The following R packages were used: ‘dplyr’^45^, ‘tidyr’^46^, ‘car’^47^, and ‘ggplot2’^48^.

## Results

### Characterization of trace element concentrations in soil and agricultural crops

**Fig. 3:**
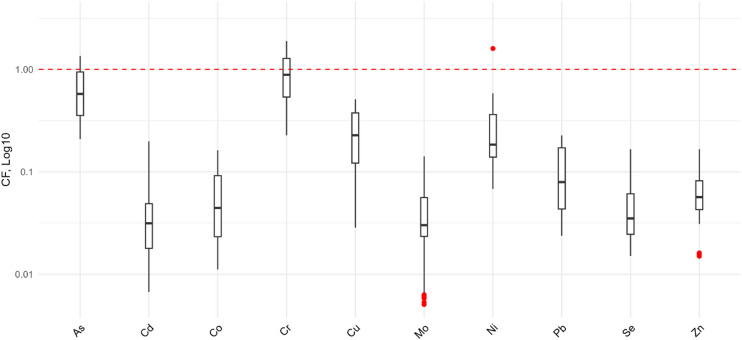
Distribution of Contamination Factors (CF, log_10_) for trace elements in soil, relative to the prevention value (PV) by CONAMA^38^. Boxes show dispersion; red dots are outliers; the red dashed line at y = 1 indicates the PV threshold.

The descriptive statistics of TEs from 84 soil samples and their respective reference values are presented in Table 3. TEs concentrations in soil varied widely across the analyzed locations, with Cr showing the lowest variation and Cd the highest. The boxplot results (Fig. 3) provide an overview of data distribution, highlighting exceedances of the PV (indicated by a red reference line), as well as identifying variation patterns and anomalous concentrations among samples.

**Table 3:** Descriptive analysis results of trace elements in Mato Grosso soils (mg kg^−1^ except for Fe) and reference values.

Element	Mean	Median	Minimum	Maximum	CV (%)	PV	IV	QRV
**As**	9.95	8.42	3.13	20.31	49.9	15	35	7.60
Cd	0.05	0.04	0.01	0.26	86.5	1.3	3	0.87
Co	1.56	1.06	0.28	4.06	75.1	25	35	–
Cr	69.74	66.98	17.02	141.91	44.4	75	150	52.73
Cu	14.23	13.61	1.71	30.63	58.5	60	200	16.50
Fe (g kg^− 1^)	44.89	42.20	13.67	83.47	48.4	–	–	2.31
Mn	84.9	68.9	14.4	314.8	74.2	–	–	351.0
Mo	1.26	0.89	0.15	4.25	77.5	30	50	–
Ni	7.56	5.49	2.04	47.99	79.5	30	70	8.86
Pb	7.72	5.71	1.71	16.29	63.0	72	180	13.42
Se	0.24	0.17	0.08	0.83	74.5	5	–	–
Zn	20.21	16.98	4.50	49.91	53.5	–	450	29.24

CV, coefficient of variation; QRV, quality reference value (90th percentile)^28^; PV, prevention value; IV, investigation value, according to Conama^38^.

The analyses revealed that the concentrations of some elements exceeded the reference values, particularly As and Cr. These two elements surpassed both the proposed QRV and the PV, with approximately 54% and 67% of the analyzed samples exceeding the QRV, while 17% and 42% exceeded the PV, respectively. Additionally, some elements exceeded only the QRV. Fe had the highest percentage of samples above this limit, with 81.0%, followed by Cu (41.7%), Ni (32.1%), Pb (22.6%), and Zn (20.2%). In contrast, Cd, Co, Mn, Mo, and Se did not exceed any of the established limits.

The descriptive statistics of TEs in 84 soybean samples, 48 corn samples, 12 popcorn samples, and 8 pasture samples are presented in Table 4, along with the legal limits established by Anvisa^39^. The Brazilian limits are close to global standards ^40,49^. According to the Codex Alimentarius^40^, the Cd limit is 0.1 mg kg^−1^ for both legumes and cereal grains, while the Pb limit is 0.1 mg kg^−1^ for legumes and 0.2 mg kg^−1^ for cereal grains. Regarding pasture, leafy vegetables can serve as a reference category, with limits of 0.2 and 0.3 mg kg^−1^ for Cd and Pb, respectively. Based on national and international standards, the concentrations of As, Cd, and Pb were within safe limits, except for one Pb sample in corn, demonstrating that these foods are safe for consumption.

**Table 4:** Descriptive analysis of trace elements in agricultural crops from Mato Grosso and Brazilian legal limits^39^ in mg kg^−1^.

Element	Crop	*N*	Mean	Median	Minimum	Maximum	CV %	Legislação
As	Corn	48	0.0007	0.0005	< DL	0.008	156.6	0.3^1^
	Popcorn	12	0.0006	0.0006	< DL	0.0008	28.5	0.3^1^
	Soybean	84	0.0020	0.0017	0.0010	0.0090	66.8	0.1^2^
	Pasture	8	0.058	0.043	0.020	0.11	59.2	0.3^3^
Cd	Corn	48	0.0006	0.0005	< DL	0.00205	65.0	0.05
	Popcorn	12	0.0009	0.0008	0.0006	0.00178	33.7	–
	Soybean	84	0.012	0.010	0.005	0.03852	51.3	0.2
	Pasture	8	0.010	0.008	0.004	0.026	72.0	0.2^3^
Co	Corn	48	0.003	0.003	< DL	0.007	41.6	–
	Popcorn	12	0.003	0.002	< DL	0.008	57.6	–
	Soybean	84	0.07	0.04	< DL	0.24	94.1	–
	Pasture	8	0.03	0.03	0.02	0.03	21.9	–
Cr	Corn	48	0.08	0.07	< DL	0.14	25.1	–
	Popcorn	12	0.09	0.08	0.071	0.14	21.7	–
	Soybean	84	0.06	0.04	< DL	0.54	129.2	–
	Pasture	8	0.67	0.60	0.26	1.23	49.4	–
Cu	Corn	48	1.48	1.44	0.93	2.26	23.1	–
	Popcorn	12	2.02	1.82	1.08	3.04	35.6	–
	Soybean	84	9.70	9.72	7.19	12.50	13.7	–
	Pasture	8	2.41	2.11	1.58	4.60	40.8	–
Fe	Corn	48	14.26	14.48	10.20	31.94	22.8	–
	Popcorn	12	11.90	11.77	10.98	13.13	5.9	–
	Soybean	84	60.66	59.01	47.93	95.73	14.0	–
	Pasture	8	227.2	173.7	84.2	415.6	58.1	–
Mn	Corn	48	3.99	3.96	2.53	5.72	20.4	–
	Popcorn	12	5.22	4.47	3.96	7.43	25.7	–
	Soybean	84	21.82	20.03	16.23	59.72	31.5	–
	Pasture	8	11.49	8.56	6.64	22.52	48.6	–
Mo	Corn	48	0.43	0.42	0.10	0.92	55.2	–
	Popcorn	12	0.54	0.59	0.38	0.68	19.1	–
	Soybean	84	6.44	4.66	0.83	23.08	81.3	–
	Pasture	8	0.320	0.297	0.203	0.613	41.2	–
Ni	Corn	48	0.14	0.13	< DL	0.83	81.1	–
	Popcorn	12	0.09	0.08	0.07	0.11	14.1	–
	Soybean	84	0.64	0.36	0.14	2.82	96.9	–
	Pasture	8	0.48	0.46	0.28	0.72	32.1	–
Pb	Corn	48	0.03	0.02	< DL	0.17	96.6	0.1
	Popcorn	12	0.02	0.01	< DL	0.08	115.8	–
	Soybean	84	0.01	0.007	< DL	0.05	84.8	0.2
	Pasture	8	0.12	0.11	< DL	0.18	31.7	0.3^3^
Se	Corn	48	0.003	0.003	< DL	0.008	70.4	–
	Popcorn	12	0.001	0.001	0.0004	0.002	36.9	–
	Soybean	84	0.005	0.004	0.001	0.02	68.3	–
	Pasture	8	0.007	0.005	0.003	0.01	68.5	–
Zn	Corn	48	16.20	16.40	10.68	21.21	16.3	–
	Popcorn	12	18.52	18.27	17.30	20.62	6.0	–
	Soybean	84	33.72	34.45	23.42	40.76	13.4	–
	Pasture	8	14.23	11.60	8.06	34.42	60.6	–

^1^Value for cereals, ^2^Value for legumes-beans, ^3^Value for leafy vegetables—Brazilian legislation (ANVISA) does not set limits for pasture or animal feed.

### Differences between crops and locations

Among the evaluated products, the analysis of data distribution indicated differences in the order of TE concentrations among products (Table 4). Corn and popcorn showed similar profiles, with Zn being the element with the highest concentration, followed by Fe and Mn, while As and Cd had the lowest values. In soybean, Fe stood out with the highest concentration, followed by Zn and Mn, with As and Se being the least concentrated. In pasture, Fe was predominant, while Se and Cd had lower concentrations. The highest CVs were recorded for As in maize, followed by Cr in soybean and Pb in popcorn. These CVs do not directly reflect the variations in ET concentrations in the soil. This suggests that they may be influenced by soil properties, agricultural practices, physiological characteristics of the crops, and external sources such as atmospheric deposition or uptake through non-root pathways.

The Kruskal-Wallis test revealed significant differences (*p* < 0.001) in the concentration of all TEs among the evaluated crops (Table 4). Dunn’s post-hoc test showed that, between corn and popcorn, no significant difference was found for most elements, except for Se, which showed a significant difference (*p* < 0.05). Pasture exhibited significantly higher As, Cr, Fe, and Pb concentration than soybeans, corn, and popcorn (*p* < 0.05). For Cd, Co, Mn, Ni, and Se, pasture concentrated more than corn and popcorn (*p* < 0.01), with no differences compared to soybeans. Regarding Cu, Mo, and Zn, soybeans had a higher concentration than pasture (*p* < 0.01) as well as corn and popcorn (*p* < 0.001), with no significant differences between pasture, corn, and popcorn. Given the absence of significant differences between corn and popcorn, except for Se, these crops were grouped and referred to as “corn/popcorn” for the analysis of concentration variations among locations.

The same test also indicated significant differences between locations (Table 5) regarding TE levels in soil and TE concentrations in soybean and corn/popcorn grains. The significant differences identified by Dunn’s post-hoc test primarily occurred between locations with the highest and lowest TE levels, both in soil and grains.

**Table 5:** Means of trace element levels in soils and their concentrations in soybean and corn/popcorn grains for the different municipalities.

Locations element	CJ	CV	JU	LRV	PA	OS	PO	PL	RO
Soil (mg kg^− 1^; Fe in g kg^− 1^)									
As***	11.27	15.21	4.95	4.98	8.44	7.82	14.57	5.03	16.78
Cd***	0.06	0.07	0.03	0.03	0.02	0.02	0.03	0.06	0.17
Co***	0.76	1.31	0.81	0.33	1.81	3.50	3.72	0.51	2.36
Cr***	86.79	88.49	29.20	79.63	46.51	85.19	64.04	39.31	108.5
Cu***	16.61	13.87	4.90	7.43	6.10	25.03	27.03	7.87	23.95
Fe***	58.96	56.82	24.50	26.96	35.51	55.47	48.38	20.92	74.52
Mn***	67.2	98.0	37.1	26.2	61.4	78.9	193.8	37.1	194.6
Mo***	1.66	1.55	0.88	0.73	0.22	0.20	3.59	0.78	1.60
Ni***	3.80	7.35	3.11	5.40	5.40	14.14	13.61	4.52	15.65
Pb***	4.37	7.16	3.44	3.05	10.95	14.30	13.53	2.59	14.86
Se***	0.16	0.21	0.26	0.17	0.11	0.14	0.68	0.12	0.35
Zn***	23.30	27.57	9.31	12.78	13.15	17.53	17.12	18.00	44.03
Soybean (mg kg^− 1^)									
As***	0.002	0.002	0.002	0.001	0.005	0.002	0.002	0.002	0.002
Cd***	0.011	0.010	0.010	0.006	0.011	0.015	0.008	0.012	0.023
Co***	0.13	0.02	0.03	0.04	0.05	0.17	0.13	0.03	0.02
Cr*	0.04	0.05	0.03	0.05	0.24	0.04	0.05	0.04	0.04
Cu***	9.74	10.70	9.55	8.00	9.39	10.39	9.97	8.34	10.77
Fe***	61.57	57.70	56.27	52.10	81.41	58.79	61.82	55.89	63.60
Mn***	19.42	17.80	19.52	20.48	19.88	21.83	38.69	20.79	22.35
Mo***	10.96	4.87	11.10	3.68	1.34	14.25	4.12	1.34	2.52
Ni***	0.81	0.30	0.31	0.42	0.83	1.20	1.60	0.35	0.21
Pb***	0.008	0.02	0.01	0.01	0.01	0.01	0.02	0.01	0.01
Se***	0.003	0.006	0.009	0.002	0.002	0.003	0.007	0.006	0.009
Zn***	30.71	34.62	31.15	38.06	29.18	30.07	36.12	38.11	37.79
Corn/Popcorn (mg kg^− 1^)									
As***	0.0006	0.0005	0.0005	0.0004	0.0008	–	–	0.0014	0.0007
Cd***	0.0009	0.0004	0.0006	0.0007	0.0003	–	–	0.0004	0.0009
Co***	0.003	0.002	0.004	0.002	0.003	–	–	0.003	0.003
Cr*	0.09	0.08	0.08	0.07	0.07	–	–	0.08	0.09
Cu***	2.02	1.60	1.27	2.10	1.23	–	–	1.34	1.48
Fe***	11.90	11.93	15.31	11.26	15.12	–	–	15.41	14.82
Mn***	5.22	4.20	4.26	3.39	3.08	–	–	3.75	5.27
Mo***	0.54	0.45	0.73	0.58	0.18	–	–	0.16	0.37
Ni***	0.09	0.07	0.14	0.09	0.23	–	–	0.14	0.14
Pb***	0.02	0.02	0.03	0.01	0.02	–	–	0.03	0.03
Se***	0.001	0.003	0.004	0.001	0.0004	–	–	0.004	0.006
Zn***	18.52	14.08	16.39	17.32	12.44	–	–	16.32	19.48

Locations: CJ, Campo de Júlio; CV, Campo Verde; JU, Juscimeira; LRV, Lucas do Rio Verde; PA, Paranatinga; PS, Planalto da Serra; PO, Poconé; PL, Primavera do Leste; RO, Rondonópolis. According to the Kruskal-Wallis test, *** indicates high significance (*p* < 0.001) and * indicates moderate significance (*p* < 0.05).

Based on the analysis of soil data distribution, Rondonópolis and Poconé stand out for having the highest concentrations of several elements, such as As, Cu, Mn, Ni, Pb, and Se. On the other hand, Juscimeira, Lucas do Rio Verde, and Primavera do Leste frequently recorded the lowest concentrations. Regarding concentrations in soybean grains, similar to the soil, Rondonópolis showed the highest concentrations of As, Cd, Cu, Fe, and Se, while Poconé stood out for the highest concentrations of Co, Cu, Mn, Ni, and Se. Conversely, Juscimeira recorded the lowest concentrations of Cd, Co, Cr, and Mn; Lucas do Rio Verde had the lowest levels of As, Cu, Fe, and Se; and Primavera do Leste had the lowest Fe, Mo, and Pb concentrations. However, in Rondonópolis, an inverse pattern was observed for Ni and Pb, as their concentrations in grains were lower despite their higher soil levels. In corn/popcorn, Rondonópolis had the highest concentrations of Cd, Cr, Mn, Zn, and Se, whereas Lucas do Rio Verde exhibited the lowest concentrations of As, Co, Fe, Mn, Mo, and Pb, mirroring soil levels. However, this crop showed a different behavior: despite the lower soil contents, Primavera do Leste stood out with higher concentrations of As, Fe, Pb, and Se, while Juscimeira also presented higher concentrations of Co, Fe, Mo, and Pb.

These results indicate a trend in which grain concentrations reflect soil levels. However, Spearman’s correlation analysis between soil levels and grain concentrations revealed that strong correlations were mostly absent, with relationships predominantly weak to moderate. Notably, a moderate correlation was observed between Se in soybean and soil (*r* = 0.59) and between Zn in corn and soil (*r* = 0.61).

### Influence of different environments and correlations between trace elements and macronutrients in the soil

The results showed that TE levels varied significantly across different environments - lithology, soil type, and geological province (Table 6). In general, the highest TE levels were associated with metamorphic lithology, Red and Yellow Latosols, and the Tocantins province, while the lowest levels were found in soils associated with sedimentary and igneous- sedimentary lithologies, Neosols, and the Cenozoic province.

**Table 6:** Mean values (mg kg^−1^ except for Fe) of trace element levels in the soil across different environments - lithology, soil type, and geological province.

(*n*)	Lithology					Soil type					Province		
		Sdc (16)	Sd (52)	Mt (8)	Is (8)		RYL (12)	RL (48)	CA (16)	NE (8)		Cen (60)	Toc (24)
As	***	11.19	9.67	14.57	4.98	***	15.55	10.03	8.13	4.98	ns	9.86	10.28
Cd	***	0.11	0.04	0.03	0.03	***	0.05	0.07	0.02	0.03	***	0.07	0.02
Co	***	1.48	1.47	3.72	0.33	***	2.13	1.28	2.65	0.33	***	1.00	3.01
Cr	Ns	73.50	67.57	64.04	79.63	Ns	81.33	66.09	65.85	79.63	ns	71.22	65.25
Cu	***	15.72	13.01	27.03	7.43	Ns	17.21	14.35	15.57	7.43	**	12.31	19.39
Fe	Ns	50.69	45.45	48.38	26.96	Ns	54.01	45.54	45.49	26.96	ns	44.38	46.45
Mn	***	120.2	66.9	193.8	26.2	***	125.8	90.04	70.15	26.16	**	74.83	111.4
Mo	***	1.22	1.00	3.59	0.73	***	2.09	1.49	0.21	0.73	*	1.23	1.34
Ni	**	9.83	6.37	13.61	5.40	***	9.08	6.92	9.77	5.40	***	6.26	11.05
Pb	***	9.22	7.19	13.53	3.05	***	9.39	6.56	12.62	3.05	***	5.73	12.93
Se	***	0.32	0.16	0.68	0.17	**	0.33	0.27	0.13	0.17	ns	0.21	0.31
Zn	*	29.40	19.10	17.12	12.78	**	22.88	22.52	15.34	12.78	ns	22.01	15.93

Sd, sedimentary; Sdc, sedimentary conglomerate; Mt, metamorphic; Is, igneous-sedimentary; CA, cambisol; RL, red latosol; RYL, red-yellow latosol; NE, neosol; Cen, cenozoic; To, tocantins.

The elements As, Mo, Se, Mn, and Ni showed higher concentrations in metamorphic lithology and Red-Yellow Latosols. Among these, Mo, Se, and Mn also stood out in Red Latosols, while Ni had higher concentrations in Cambisols, and Mo, Mn, and Ni were more elevated in the Tocantins province. However, As and Se were not explained by province variations. The elements Co and Pb, along with Ni, were associated with Cambisols, metamorphic lithology, and the Tocantins province. On the other hand, Cu, despite not being influenced by soil type, exhibited the highest concentrations in metamorphic lithology and the Tocantins province. In contrast, Cd and Zn showed higher concentrations associated with sedimentary conglomerate lithology and Red-Yellow and Red Latosols. Cd also exhibited higher concentrations in the Cenozoic province. Meanwhile, Cr and Fe did not show any correlation with the analyzed environmental variations.

**Fig. 4:**
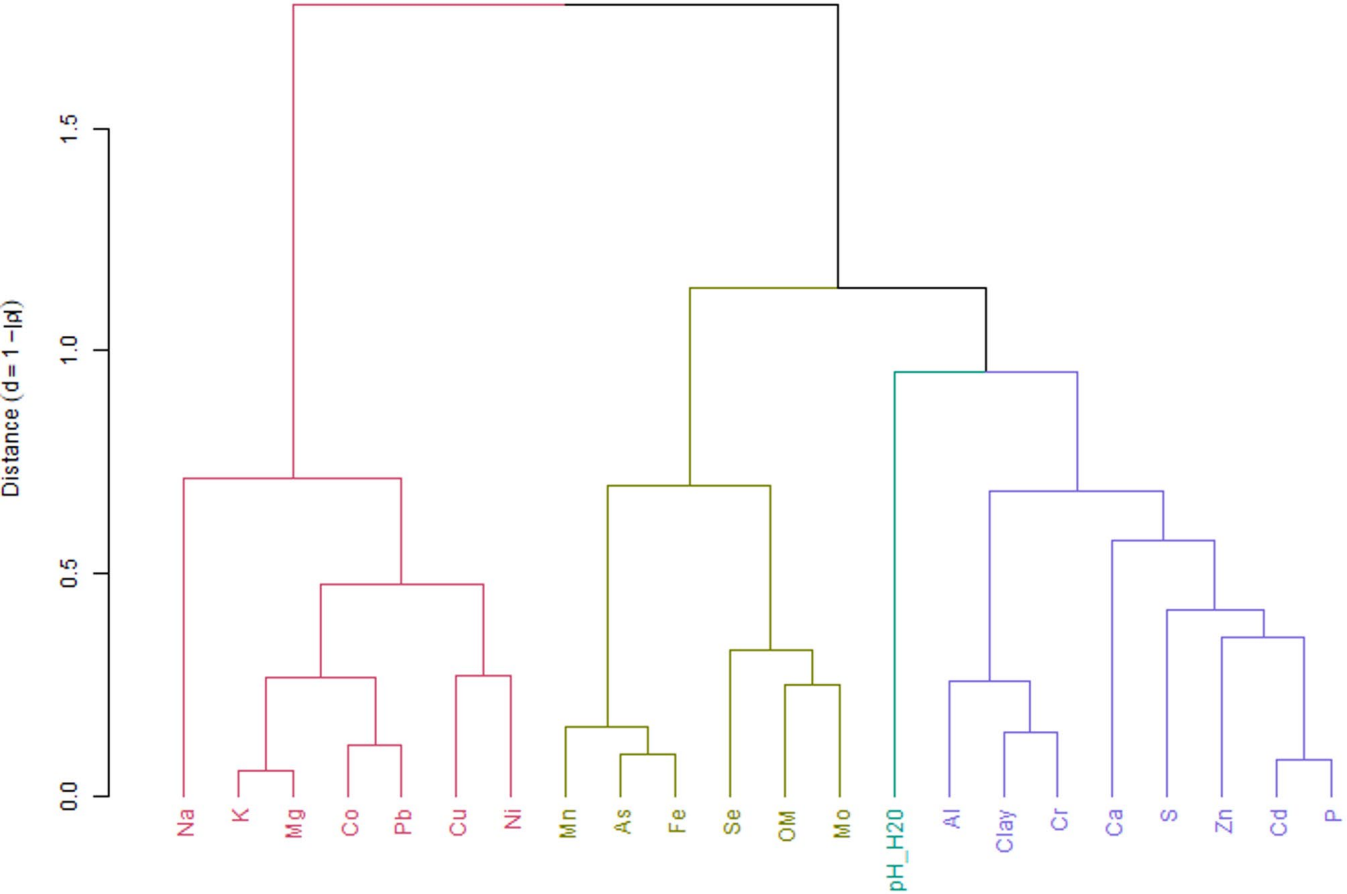
Dendrogram of cluster analysis for trace elements and macronutrients. Clustering was performed using the Ward.D2 method based on a distance matrix (d = 1 − |ρ|) derived from Spearman’s correlation. Colors facilitate the identification of the four groups formed based on correlation similarity. Organic Matter (OM).

These results are consistent with the correlation dendrogram (Fig. 4), which highlighted the formation of three main groups of elements with similar contents. The first group included Co, Pb, Cu, and Ni (associated with the Tocantins province), the second grouped As, Fe, Mo, and Se (related to metamorphic lithology) - including organic matter, while the third was composed of Cr, Zn, and Cd (linked to sedimentary lithology)—including clay. These groupings reinforce the relationships observed among the elements and the studied environments, evidencing similar patterns in their distribution across different soil types and lithologies.

The influence of these different environments was evident in the distribution of TEs among the studied locations (Table 5). In Poconé, the highest concentrations of As, Co, Cu, Mn, Mo, Ni, Pb, and Se corresponded to metamorphic lithology, and - except for As and Se - also to the Tocantins geological province, indicating a strong contribution from regional geology. In Planalto da Serra, the predominance of Cambisols and the Tocantins geological province was determinant for the concentrations of Co, Ni, and Pb. In this location, Cu concentrations were attributed exclusively to the Tocantins province. In Campo Verde, the higher concentrations of As and Cd were attributed to the presence of Latosols, with Cd being linked exclusively to the Cenozoic province. In Rondonópolis, the highest concentrations of Cd and Zn were associated with sedimentary conglomerate lithology and Red Latosols. Mn was related to Red Latosols, Co to Cambisols, while As, Cu, Ni, and Pb were not associated with any specific environmental factor.

### Bioaccumulation factor

**Fig. 5:**
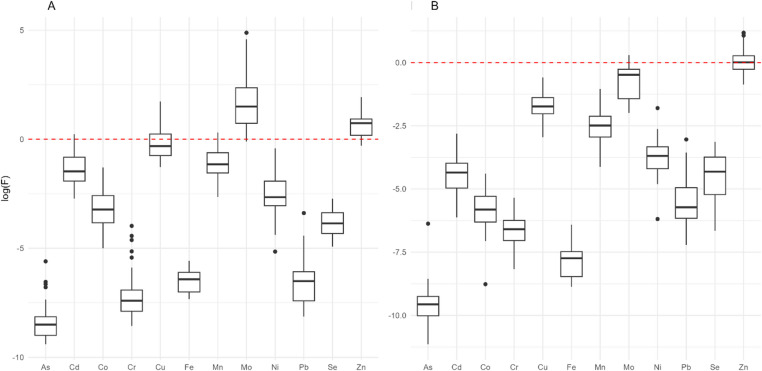
Comparison of Bioaccumulation Factors (BAF) of the elements using Welch’s ANOVA, with a significance level of 5% (*p* < 0.05). The figure shows (**A**) soybean and (**B**) corn/popcorn, with the red line indicating the reference value for high accumulation.

The transfer efficiency of elements from soil to grains, measured by the bioconcentration factor (BCF), indicated higher concentrations in grains for some TEs (Fig. 5), classified as high, very high, and hyperaccumulation. However, most elements showed low to very low accumulation, with some exceptions. Cd and Mn concentrations in soybean grains were predominantly low to moderate, with four samples classified as high accumulation (BCF > 1) for each element, observed in Planalto da Serra for Cd and in Juscimeira for Mn. Cu accumulation in soybean ranged from low to high across all locations, except in Campos de Júlio and Poconé, where no high accumulation was recorded. Mo in maize/popcorn ranged from moderate to high accumulation, and in soybean from high to very high, with all samples from Planalto da Serra classified as hyperaccumulation. Zn, in both crops, was predominantly classified as high accumulation, except in Rondonópolis, where maize/popcorn showed low accumulation and soybean, moderate.

## Discussion

### Trace element contents in soil and agricultural products

Cr and As contents exceeded the VP^38^ and VRQ^28^ values, indicating possible alterations in soil quality according to Brazilian legislation. However, these values may be associated with natural concentrations, since the state of Mato Grosso includes regions with naturally elevated levels of these elements, including Fe^27^. In this study, Fe showed a high percentage of samples exceeding the QRV, but there is no PV established for this element. The mean As concentration (9.95 mg kg^−1^) is close to natural background levels reported for Europe^50^, the United States^51^, and Minas Gerais, Brazil^52^. The mean Cr concentration (69.74 mg kg^−1^) is similar to levels reported in Minas Gerais^52^ and Rio Grande do Sul^53^, while the mean Fe concentration (44.89 g kg^−1^) is comparable to values found in Paraíba, Minas Gerais, and Goiás^54^.

Besides Fe, a smaller percentage of samples showed Cu, Ni, Pb, and Zn contents exceeding the VRQ, which may be related to both natural sources and other anthropogenic inputs. Binde et al.^27^ reported higher levels attributed to natural sources, and the averages of these elements fall within the lower range of natural values in the country^54–56^. However, the possible contribution of agricultural practices cannot be ruled out, especially the application of phosphate fertilizers, which may contain TE impurities^23,57,58^. In the case of Zn, for example, although its natural concentrations in Brazilian soils are generally low - justifying its supplementation in many regions^59^- this practice may contribute to localized increases in the levels observed in some samples.

Regarding the analyzed crops, despite the naturally high As concentrations in some regions of the state, the concentrations of this element, as well as those of Cd and Pb, remained below the established food safety limits. In corn, similar to popcorn, the concentrations of the analyzed elements were generally lower than those reported in other studies, except for Mo, which had higher concentrations^17,60–64^. In soybeans, the same pattern was observed^3,12,63,65^. In the pasture, Cr showed an intermediate value, while the other elements, except Mo, exhibited concentrations lower than those reported in the literature^14,66–68^. For Mo, the values fall within the typical range for grasses, as described by Suttle^69^.

Regarding Fe, although a high percentage of samples showed concentrations above the QRV in the soil, Brazilian legislation does not establish a VP for this element, nor does it define maximum allowable limits in food. According to the United States National Institutes of Health^70^, the tolerable upper intake level (UL) of Fe for adults is 45 mg day^−1^, indicating that the levels found in this study are far below concentrations that could pose a toxicological risk. Therefore, considering that Fe is an essential micronutrient for human health, its presence in grains can be seen as positive from a nutritional perspective.

However, it is noteworthy that Fe concentration in soybeans can vary significantly depending on genotype, edaphoclimatic conditions, and management practices. In the present study, concentrations ranged from 47.93 to 95.73 mg kg^−1^, which are lower than those reported by Oliveira et al.^71^, who found values between 58 and 163 mg kg^−1^ in greenhouse-grown plants. This discrepancy may be attributed to differences in environmental growing conditions as well as genetic variability among the evaluated materials.

For animal health, although Fe is also essential, excess levels can interfere with the absorption of other elements such as Cu, Zn, and Mn. In this study, pasture samples (*Urochloa ruziziensis*) showed Fe concentrations ranging from 84.2 to 415.6 mg kg^−1^, where higher values were associated with greater soil concentrations of the element. These values fall within the range reported for pastures (18−1,000 mg kg^−1^), according to Kabata-Pendias^72^, and are consistent with the levels of 25.71 to 1,230.79 mg kg^−1^ observed by Darch et al.^73^ in different forage species. This indicates that there was no excessive accumulation of Fe in the pastures, although values approaching the upper limit warrant attention in livestock production systems, considering that the maximum tolerable level (MTL) is 500 mg kg^−1^^74^. The observed variation reflects edaphic influences, reinforcing the need for monitoring in naturally Fe-rich soils to prevent nutritional imbalances and ensure safety within the food chain. However, the low analytical recovery of Fe in this matrix introduces uncertainty and may lead to underestimation of actual concentrations, and should be considered in the assessment of animal health risk.

### Different agricultural products, locations, and influences of environmental factors and correlations among trace elements

Essential elements such as Fe, Zn, and Mn were more readily absorbed due to their fundamental physiological roles in plant growth and development, whereas elements like As, Cd, and Se, which have no specific function, were less absorbed^8,75^. Therefore, the uptake of TEs by plants may occur according to their nutritional requirements, but it is also influenced by factors such as availability, ionic competition, and element speciation. However, differences in the export of these elements were observed among the analyzed species, possibly due to genetic variations^8^. The absorption, translocation, and accumulation of TEs vary depending on the plant species and the type of TE involved^6,62,75^. Crops such as soybeans and corn exhibit significant differences in these processes due to their physiological requirements and characteristics^76^.

Soybeans, for instance, have a high protein content and require micronutrients such as Fe, Zn, and Cu, which are essential for protein synthesis^76^. Their dense and branched root system enhances nutrient absorption^77^. In contrast, corn employs more efficient exclusion and root sequestration mechanisms, reducing the translocation of TEs to the grains, thus minimizing food contamination risks^8,78^. While corn can effectively absorb TEs from the soil, their transport to the grains is limited^8^. Additionally, some grasses, such as Brachiaria, exhibit high tolerance and even hyperaccumulation of specific TEs, thus being more efficient in retaining Cd and Pb in their tissues^79,80^. The highest concentrations of most TEs were observed in pasture, except for Cu, Mo, and Zn. These elements had higher concentrations in soybeans, possibly due to fertilization - evidenced in the bioaccumulation analysis presented in the following section.

Regarding TE levels in the soil, the highest concentrations were found in Poconé and Planalto da Serra. These were attributed to environmental factors, primarily metamorphic lithology and the Tocantins province. The presence of mafic terrains in this province^81^ may contribute to increased TE levels in the soil^82–84^, in contrast to sedimentary environments^85,86^. The influence of metamorphic lithology and the Tocantins province on Mn, Ni, Mo, and Cu levels was also noted by Silva et al.^28^, and Binde et al.^27^. In these locations, variations in ET concentrations in the crops were observed, highlighting variations in the availability, uptake, and translocation of these elements (Jordan-Meille et al. 2021). In Poconé, only Mn and Ni had higher concentrations in soybeans, while in Planalto da Serra, Co, Cu, and Ni were reflected in the crop.

In Rondonópolis and Campo Verde, the higher soil TE contents are related to the characteristics of the local soil types, reflecting pedogenetic processes such as weathering, leaching, and adsorption^87,88^, with no evidence of a direct influence from the parent material. The absence of a direct correlation with the parent material suggests that agricultural practices may be contributing to the elevated TE levels in these locations. However, it is important to consider that pedogenetic processes concentrate more stable elements, such as Fe and Al, while the more mobile ones are removed^87,88^. Although the lack of correlation with the parent material suggests a possible anthropogenic contribution to the elevated soil TE contents, a natural origin cannot be ruled out. Therefore, a more detailed study is necessary to better understand these processes at the local scale.

Crop responses varied in these locations. In Rondonópolis, the highest Cd levels in the soil were reflected in higher concentrations of this element in soybeans, as well as As, Cu, and Fe. In Campo Verde, however, no correlation was observed between TE levels in the soil and plant concentrations, suggesting that absorption was limited or that other factors interfered with TE availability for the crop. These results indicate that the interaction between geological, pedological, and agricultural factors strongly influences TE distribution and absorption in crops. Understanding these factors is essential for assessing soil quality and the impact of TEs on agricultural production, enabling more sustainable and safer management practices.

### Bioaccumulation factor

Although the elements As, Cr, and Fe showed elevated levels in the analyzed soils, their transfer efficiency to the grains was low, suggesting low soil availability and/or barriers to translocation^17,89,90^. These barriers are evident in the differences in concentrations observed among the analyzed crops^91,92^. Low bioavailability may be related to soil interactions, including adsorption onto Fe and Al oxides and pH variations, which influence element mobility^89,93^. Excess Fe in the soil may contribute to the retention of As and Pb, reducing their availability and, consequently, their translocation to grains^93^. In this study, the highest As levels were found in Oxisols, which have high contents of iron oxides (goethite, hematite, and maghemite) and gibbsite^33^.

Among non-essential TEs, Cd exhibited the highest soil-to-grain transfer efficiency, possibly due to its divalent chemical form (Cd^2+^), which is similar to essential elements, facilitating its absorption and translocation within plant tissues^89,91,94^. In addition, Cd tends to be more mobile under lower pH conditions^72^, and the pH in the studied areas ranged from 5.2 to 6.7, which may have contributed to its increased mobility and uptake by plants. Regarding Cd, Cu, Zn, and Mo, which had BFA values greater than 1, there is evidence that their source, at least partially, may be linked to foliar application or another type of fertilization applied after soil sampling. This situation was observed specifically for Cd in soybean crops in Planalto da Serra, indicating the need for a more detailed evaluation regarding the adequacy of local agricultural practices. Additionally, it is important to consider that Cd is frequently cited as an impurity in phosphate^57,95^ and organic fertilizers^96^.

For Cu, this situation was more widespread, occurring exclusively in soybean crops. In the case of Zn and Mo, a similar pattern was observed, but these elements were also present in corn crops. Foliar Zn application has been recommended even when soil concentrations are above the critical level, as it can enhance crop yield. Additionally, for elements such as Ni, Mo, Co, Cu, Mn, and B, which are frequently deficient in the Cerrado, supplementation is recommended every two growing seasons^97^. Mo, in particular, plays a key role in biological nitrogen fixation (BNF), a widely used technique in Brazil for legume production. Mo acts as a cofactor for the nitrogenase enzyme, which converts atmospheric N_2_ into forms assimilable by plants. Therefore, technical recommendations suggest Mo supplementation, and foliar applications are indicated to increase Mo content in soybean grains^98,99,100^.

## Conclusions

The concentrations observed in this study, both in soils and agricultural products, were mostly below reference values for TEs, suggesting no immediate risk to agricultural production when assessed against current regulatory limits. Despite the occurrence of elevated As, Cr, and Fe levels in soils, transfer to grains was limited under the evaluated conditions, indicating a low immediate risk of food contamination. e showed wide variation in pastures, with values close to the maximum tolerable level for animals. However, these results are associated with analytical uncertainties due to low recovery, reinforcing the need for continuous monitoring and appropriate management of mineral supplementation. The integration of pedological and geological information with statistical analyses provided support for the discussion of possible trace-element sources. Despite the lack of detailed site management histories and direct analyses of agricultural inputs, the observed patterns were generally consistent with agricultural practices widely adopted in the state and should therefore be interpreted as inferential. Cd may be primarily associated with phosphate fertilization, among other possible sources, whereas Mo is possibly related to biological nitrogen fixation. Mn, Cu, and Zn, in turn, are associated with the need for localized fertilization, with Zn also correlated with the phosphorus source. In general, agricultural production in the state does not present an immediate risk. However, future studies are needed on the sources and distribution of trace elements along the soil profile, as soil acidity enhances their vertical mobility. The results highlight the complexity of trace element transfer and accumulation processes and the role of agricultural practices in their dynamics.”

## Data Availability

The datasets generated and/or analyzed during the current study are not publicly available due to their size and institutional data management restrictions but are available from the corresponding author on reasonable request.
